# Precise Probing of Interfaces at the Single-Molecule Scale

**DOI:** 10.3390/nano16100573

**Published:** 2026-05-07

**Authors:** Enyu Zhang, Zhiping Chen, Shuai Wang, Cong Zhao, Hongyu Ju, Yingze Sun, Chuancheng Jia

**Affiliations:** Center of Single-Molecule Sciences, Institute of Modern Optics, Frontiers Science Center for New Organic Matter, Academy for Advanced Interdisciplinary Studies, College of Electronic Information and Optical Engineering, Nankai University, 38 Tongyan Road, Jinnan District, Tianjin 300350, China

**Keywords:** single-molecule junctions, interfacial physicochemistry, in situ monitoring, solid–liquid interfaces, micro/nano materials, dynamic processes

## Abstract

The macroscopic functionality of nanoscale systems is fundamentally governed by microscopic physicochemical processes at material interfaces. However, conventional ensemble-averaged characterization techniques often obscure these subtle interfacial nuances due to their inherent limitations in spatial and temporal resolution. This review examines how single-molecule electrical measurements overcome these constraints by acting as precise analytical probes that directly transduce interfacial events into quantifiable conductance signals. By summarizing recent advances, we demonstrate how this approach resolves key physical interfacial characteristics, including distinct bonding motifs, steric configurations, and electronic coupling. We further summarize the real-time chemical interrogation of solid–liquid boundaries, which enables the capture of covalent bond formation kinetics, the dissection of catalytic reaction mechanisms, and the tracking of dynamic ion adsorption and proton transfer. Collectively, these investigations reveal interfaces as active, dynamically responsive physicochemical environments rather than simple passive structural boundaries. Finally, we propose that when employed primarily as high-resolution diagnostic tools rather than standalone electronic components, single-molecule junctions bridge atomic-scale interfacial mechanisms with macroscopic material performance, thereby providing an essential mechanistic foundations for the rational design of functional nanointerfaces.

## 1. Introduction

The macroscopic physicochemical properties of nanoscale functional systems are largely governed by the local microscopic structure at their interfaces [1,2]. Atomic arrangements, chemical bonding configurations, and electronic coupling strengths at these interfaces collectively dictate critical properties such as charge transport efficiency, catalytic activity, and device stability [3,4,5,6]. While traditional characterization techniques, including spectroscopy and electrochemistry, have long provided valuable ensemble-level insights into interfacial behavior, these ensemble-averaged measurements inherently obscure heterogeneous interfacial processes and mask the underlying mechanistic details. At the same time, vibrational spectroscopies, electrochemical methods, and scanning probe microscopy have, respectively, provided chemical-bond-level, thermodynamic, and atomic-scale structural insight into buried interfaces; multimodal approaches integrating complementary probes with theory are now aiming for predictive cross-scale descriptions [7,8]. However, these techniques do not typically offer simultaneous, real-time electrical readouts that continuously follow individual interfacial events at the single-molecule level under ambient or operando conditions. Consequently, researchers have expanded their focus beyond macroscopic bulk characterization to encompass molecular-scale investigations capable of capturing subtle structural and electronic nuances [9,10].

The advent of single-molecule junction (SMJ) technology has introduced a novel paradigm for probing micro/nano interfaces [11]. By employing techniques such as mechanically controllable break junctions (MCBJs) [12], scanning tunneling microscope break junctions (STM-BJs) [13], and conductive atomic force microscope break junctions (CAFM-BJ) [14], individual molecules can be precisely anchored between two nanoelectrodes to form well-defined SMJs [15]. This technique is particularly advantageous for interfacial science for two reasons. First, this configuration places the contact region directly within the measurement pathway, thereby enabling precise characterization of the molecule–metal contact interface [16]. In this configuration, the contact interface emerges as the pivotal region governing heterogeneous charge transport, where even minute perturbations can induce pronounced shifts in electrical characteristics [7,17,18]. Second, a defining advantage of the SMJ technique in interfacial science lies in its capacity for in situ resolution of subtle dynamic processes [19]. Real-time conductance monitoring provides high-resolution, direct access to elementary interfacial processes, including individual bond formation and rupture, molecular conformational transitions, dynamic ionic adsorption, and proton transfer kinetics [20], thereby resolving microscopic mechanisms that remain obscured by the ensemble averaging inherent in conventional techniques. From this vantage point, metal electrode interfaces are redefined not merely as passive conduits for charge transport, but as active sites participating in interfacial electronic coupling and chemical transformations [21,22]. Thus, by treating the interface as a dynamic, chemically active region, the intricate influences of local electric fields, electrical double layer structures, and ionic microenvironments on solid–liquid interfacial processes can be systematically deconstructed at the single-molecule level [23,24,25]. Experimentally, this platform measures conductance down to 10^−5^ *G*_0_ with a resolution of the same order, enabled by low-noise transimpedance amplifiers [26,27,28]. STM-BJ provides at least a millisecond time resolution, typically sampling up to 20 kHz and holding junctions for ∼200 ms. It acquires thousands of traces to produce statistically robust conductance histograms, thereby capturing transient states on shorter timescales and reversible optoelectronic switching [29]. These quantitative capabilities provide a reliable technical foundation for the identification of interfacial contact motifs, the probing of steric configurations, and the monitoring of dynamic chemical processes discussed in the following sections. Previous reviews have provided a thorough account of SMJs in the context of molecular electronics and device performance [1,2,30,31]; here, we focus on their use as probes for interfacial physicochemistry.

This review highlights the unique advantages of SMJs as a platform for probing micro/nano interfacial processes, focusing specifically on their application in deciphering microscopic physicochemical phenomena at interfaces. By systematically summarizing recent breakthroughs, we aim to present a fresh perspective on micro/nano interface science at the single-molecule scale. The first section of the review demonstrates the capability of SMJs in precisely identifying interfacial contact motifs, quantitatively assessing steric hindrance effects, and elucidating electronic coupling mechanisms. The second section emphasizes their capacity for real-time monitoring of dynamic chemical processes, detailing how electrical signals can be utilized to track in situ covalent bond formation, dissect complex reaction pathways, and quantitatively resolve ionic dynamics at solid–liquid interfaces. These advances are driving interfacial research toward the single-molecule regime. Furthermore, we propose multimodal techniques and AI-driven high-throughput analysis to unravel the nanoscale interfacial physicochemical nature and facilitate the rational design, function prediction, and practical application of functional nanomaterials.

## 2. Characterization of Physical Interfacial Properties

Elucidating the microscopic physical characteristics of material interfaces is essential for understanding nanoscale behaviors. In confined interfacial regions, strong chemical bonds provide rigid mechanical connections, whereas non-covalent interactions construct dynamic microenvironments that underpin self-assembly and molecular recognition [32,33,34,35,36]. However, ensemble measurements are inevitably averaged over heterogeneous contact geometries, making it difficult to correlate specific bonding motifs, steric arrangements, or electronic coupling strengths with charge transport properties.

SMJ techniques provide a unique platform for resolving structure-property relationships at the single-molecule level. By constructing SMJs, researchers can manipulate and characterize electron tunneling properties at the individual molecule scale, thereby enabling independent investigations of discrete non-covalent interactions under highly controlled conditions [30,31]. This capability for precise control allows researchers to probe deeply into how non-covalent forces dynamically influence the physical properties of interfaces.

Currently, single-molecule electrical characterization methods are flourishing, and have evolved into diverse interfacial probing approaches including conductance measurements, mechanoelectrical coupling, and dynamic modulation [37,38,39,40,41,42,43]. These methods demonstrate tremendous potential in revealing novel interfacial non-covalent interactions, resolving interfacial electronic coupling states, and assessing the dynamic evolution of interfacial conformations [44,45,46,47]. More importantly, through refined analysis of interfacial flexibility and conformational stability, researchers can achieve deeper comprehension of complex interfacial electronic behaviors [48]. A critical challenge in understanding interfaces lies in the intrinsic heterogeneity of interfacial structures and dynamic fluctuations in contact configurations [49]; yet SMJs can overcome this by statistically analyzing single-event conductance trajectories to reveal diverse bonding pathways and energy dissipation [50].

This chapter focuses on how SMJs serve as unique physical platforms for resolving interfacial properties at the micro- and nanoscales, emphasizing three critical dimensions. First, we introduce the use of single-molecule electrical fingerprints to precisely identify interfacial contact modes, elucidating the microscopic origins and dynamic evolution principles of non-covalent interfaces. Second, we demonstrate the technique’s spatially resolved capabilities in probing interfacial steric effects. Finally, we reveal explorations of interfacial electronic coupling mechanisms and energy level modulation behavior under different variable conditions. Through these discussions, we aim to show that SMJs represent not merely objects to be manipulated, but powerful tools for the in-depth understanding of physical processes at nanoscale interfaces.

### 2.1. Precise Analysis of Interfacial Contact

Interfacial contact modes govern charge transport efficiency and stability [7,51]. The interfacial interactions span a spectrum from strong chemical bonding to weak noncovalent forces, in which microscopic geometric configurations and their structural evolution directly modulate charge transport characteristics and environmental responsiveness. A fundamental challenge is to precisely discriminate the intrinsic attributes of distinct contact modes, decipher the structure–property relationships of noncovalent interactions within confined spaces, and understand their energy dissipation pathways, and responses to external perturbations. Addressing these challenges is essential for the functional control of interfaces.

**Figure 1:**
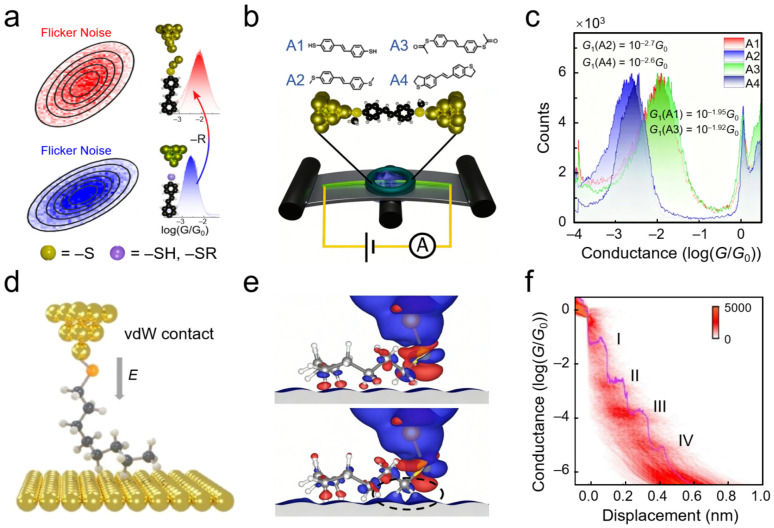
Single-molecule interrogation of interfacial bonding and van der Waals desorption. (**a**) Schematic contrast between robust chemisorption and weak physisorption. (**b**) Schematic illustration of the device architecture during mechanically controlled break-junction elongation. (**c**) Logarithmic conductance histograms resolving distinct peaks for thiol versus thioether junctions. Reproduced from Ref. [55] under a CC BY-NC-ND 4.0 license. (**d**) Device architecture for alkane/Au van der Waals contacts. (**e**) Differential charge density maps in zero field (**top**) and under a 0.5 V/Å field (**bottom**). (**f**) Representative conductance trace highlighting four discrete plateaus. Reproduced from Ref. [56] under a CC BY-NC 3.0 license.

By mapping bonding information at interfaces into quantifiable and distinguishable electrical fingerprints, researchers can resolve the essential differences among covalent, coordinate, and noncovalent interactions at the single-bond level in situ [52,53,54]. For instance, flicker noise spectroscopy is introduced to in situ probe bonding modes at gold–sulfur interfaces (Figure 1a) [55]. Using thiol and thioether derivatives sharing an identical stilbene backbone as model systems, noise power scaling indices were extracted from current signals during stretching processes using mechanically controllable break junction techniques (Figure 1b). The two molecular classes exhibited markedly distinct interfacial dynamic signatures (Figure 1c). For thiol molecules forming strong chemisorption bonds, the minimum noise scaling index was approximately 0.53, closely matching the fracture characteristics of pure metallic Au–Au atomic contacts. This reveals that during junction elongation, electrical fluctuations are dominated by the local reconstruction of gold electrode atoms rather than Au–S bond rupture. Conversely, for thioether molecules exhibiting weak physisorption, the scaling index reached 0.98. These results demonstrate the in situ discrimination of covalent and noncovalent interactions at the single-bond level.

Beyond thiol/thioether systems, single-molecule electrical platforms have been extensively applied to various anchoring groups (such as pyridine), thereby constructing a comprehensive physical picture ranging from weak noncovalent to strong covalent interactions [54]. Further investigations demonstrated that co-adsorbed molecules (such as 1-ethylimidazole) can selectively suppress Au–N interactions, providing a convenient means to modulate interfacial coupling strength [57]. These studies extend the capability from distinguishing bonding types to precisely differentiating the intrinsic attributes, such as coupling strength and chemical stability.

Building upon the discrimination of bonding types, resolving the dynamic desorption mechanisms of weak interactions requires high spatial resolution. SMJs demonstrate excellent spatial resolution and dynamic capture abilities. For example in alkyl chain systems, where interfacial connections are primarily maintained by van der Waals forces, traditional ensemble measurements struggle to distinguish specific adsorption configurations and dynamic desorption processes. However, single-molecule junction techniques can map these microscopic physical pictures into definitive electrical signals. As shown in Figure 1d, a study observed prominent dual-channel transport characteristics in conductance histograms, directly corresponding to distinct noncovalent coupling geometries between molecules and electrodes, proving that conductance signals can serve as fingerprint identifiers for interfacial contact modes [56]. Furthermore, through statistical analysis of fracture processes and calculation comparisons (Figure 1e,f); this work clarified the energy dissipation mechanisms of interfacial desorption, revealing the dynamic evolution pathways of van der Waals interfaces under mechanical stretching. In addition to alkyl chains, this van der Waals-based interfacial analysis strategy has been extended to aromatic systems. For example, systematic investigations of interactions between electrodes and acene molecules have explored correlations between van der Waals forces and interfacial coupling strength [23,58]. Collectively, these works demonstrate that single-molecule electrical platforms can transform “invisible” weak interaction physical pictures (such as adsorption configurations, desorption pathways, and interfacial stability) into clear electrical signals, providing unique experimental perspectives for understanding complex interfacial processes.

**Figure 2:**
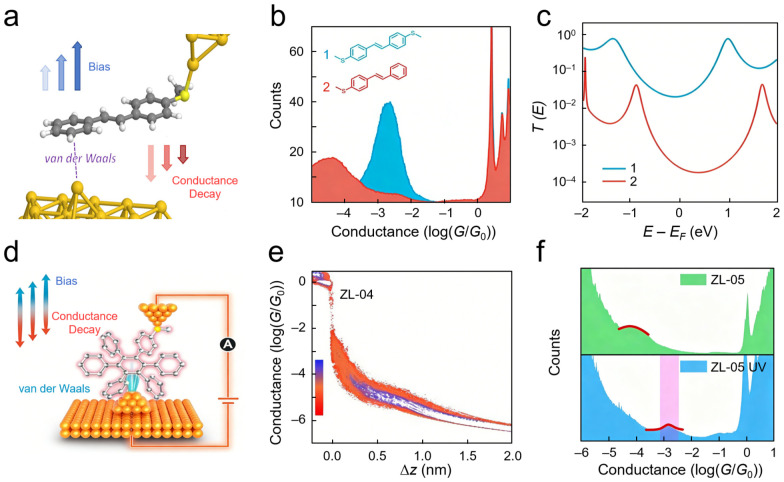
Single-molecule probing of externally regulated interfacial coupling. (**a**) Schematic of STM-BJ junction with a linker bound via van der Waals contact to gold. (**b**) One-dimensional conductance histograms for molecules 1 (blue) and 2 (red). (**c**) Calculated transmission functions for molecules 1 and 2. Reproduced from Ref. [59]. Copyright 2023 American Chemical Society. (**d**) Schematic of an STM-BJ junction featuring mixed covalent (–SMe) and noncovalent electrode contacts. (**e**) Two-dimensional conductance–displacement plot for ZL-04. (**f**) One-dimensional conductance histograms of ZL-05 before and after UV irradiation. Reproduced from Ref. [60]. Copyright 2025 Elsevier Besloten Vennootschap.

While the aforementioned studies focus on identifying the essence of interfacial contacts and mechanistic analysis, another significant advantage of SMJs lies in their ability to precisely quantify noncovalent interactions and construct stable functional interfaces. Quantitative analysis of interfacial weak interactions is crucial for understanding structure–property relationships in nanomaterials, and SMJs provide a probing platform for quantitative electrical analysis of interfacial van der Waals interactions. Traditional macroscopic characterization methods face challenges in extracting coupling parameters directly associated with geometric structures. To address this, frequency-modulated STM-BJ techniques, which apply sinusoidal modulation to electrode spacing and bias voltage combined with Fourier transform analysis of conductance responses, were employed, enabling quantitative resolution of coupling strength at SMJ interfaces (Figure 2a) [59]. By comparing molecular junctions with different connection modes using this platform (Figure 2b,c), the unique response behaviors of van der Waals-coupled interfaces under external electric fields and geometric perturbations can be directly revealed. Quantitative analysis showed that the effective conductance decay parameter *β* for molecule 1 (Au–SMe) was 0.25 ± 0.03 Å^−1^, while that for molecule 2 (Au–π vdW) was 0.63 ± 0.08 Å^−1^. As bias increased from 0.3 V to 0.9 V, *β* of molecule 2 decreased monotonically from approximately 0.6 Å^−1^ to 0.4 Å^−1^, directly quantifying the voltage-induced strengthening of van der Waals coupling. This work transforms interfacial van der Waals interactions into physical quantities that can be dynamically modulated by external fields and quantitatively measured. SMJs serve here as sensitive probes, perceiving and quantifying the microscopic evolution of interfacial weak interactions under external field action in real time, thereby offering a novel bottom-up perspective for understanding cooperative effects and response mechanisms of van der Waals interactions in macroscopic complex interfaces.

Alongside electric field modulation, mechanical force represents another crucial physical parameter for tuning van der Waals interfaces. For example, CAFM-BJ techniques were utilized to directly reveal the dynamic evolution of interfacial coupling behavior under mechanical force modulation [61]. The experiments uncovered a “self-limiting” effect of van der Waals forces on molecular configurations, providing important insights into the mechanical behavior of flexible molecules at confined interfaces. Furthermore, single-molecule conductance measurements were combined with symmetry-adapted perturbation theory to achieve the precise decomposition of van der Waals interaction components (electrostatic, induction, exchange, and dispersion energies), establishing direct structure–property relationships between molecular frameworks and van der Waals interaction components through the selective enhancement of specific interaction components via molecular skeleton design [62]. These studies collectively demonstrate that SMJs can not only “sense” the presence of van der Waals forces but also “quantify” their components, response patterns, and energy dissipation mechanisms.

Apart from quantitative characterization, noncovalent interactions provide unique pathways for constructing stable interfacial contacts and achieving external field-responsive functionalities. For instance, noncovalent molecular junction platforms utilizing π–π stacking interactions between aromatic systems were constructed to investigate the physical properties and dynamic evolution of spatially coupled interfaces [61]. This study established an interfacial contact model based on oriented aromatic ring stacking (Figure 2d), a connection mode that maintains mechanical stability while providing tunable electronic coupling channels. Electrical measurements revealed that this interfacial system maintains high conductance levels with excellent reproducibility (Figure 2e), confirming that noncovalent forces are sufficient to support stable charge transport pathways. Photo-response test data revealed the dynamic modulation mechanism of photoisomerization processes on interfacial stacking states and electronic coupling strength (Figure 2f), achieving reversible switching of interfacial transport properties. By approaching from the noncovalent interfacial coupling dimension, this work demonstrates the capability of SMJs to monitor the evolution of interfacial physical states in suit. Additionally, the influence of noncovalent interactions (such as *π*–*π* stacking and host–guest recognition) on interfacial stability has also been investigated at the single-molecule level [63,64,65].

### 2.2. Probing of Interfacial Spatial Configurations

In addition to interfacial bonding types, the spatial arrangement of molecules at metal interfaces remains equally crucial for understanding the physical properties of interfaces at micro- and nanoscales. The spatial orientation of molecular anchoring groups, steric hindrance effects of substituents, and adsorption configurations on electrode surfaces directly govern the overlap of interfacial wave functions and charge transport pathways. Resolving interfacial spatial details and mapping their structure–electronic state is crucial for understanding micro-nano structure–property relationships and for the rational design of interfacial microstructures.

**Figure 3:**
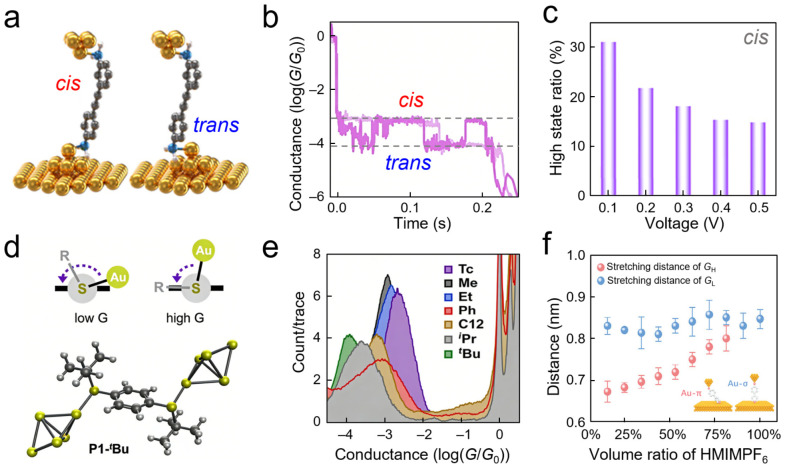
Interfacial configurations and stereoelectronic effects. (**a**) Schematic of *cis* and *trans* anchoring geometries in junctions. (**b**) Conductance–time traces and 1D histogram showing high/low conductance switching in M1 junctions. (**c**) Bias dependence of the high-conductance state ratio. Reproduced from Ref. [66]. Copyright 2024 American Chemical Society. (**d**) Newman projections of S–Au bond alignment and DFT-optimized junctions with constrained dihedral angles. (**e**) Overlaid histograms correlating thioether steric bulk with conductance decay. Reproduced from Ref. [69] under a CC BY-NC-ND 4.0 license. (**f**) Plateau length distributions of *G*_H_ and *G*_L_ states varying with ionic liquid concentration. Reproduced from Ref. [70]. Copyright 2025 John Wiley and Sons.

To this end, SMJs enable the precise identification of the spatial orientation of anchoring groups relative to electrodes and their impact on electron transmission. STM-BJ techniques were utilized to construct diphenylacetylene diamine molecular junctions; cis and trans interfacial anchoring configurations were constructed on the same rigid conjugated backbone by modulating the relative orientation of terminal amino groups with respect to gold electrodes [66] (Figure 3a). Conductance measurements revealed that these two configurations exhibited approximately an order-of-magnitude difference in conductance arising from disparities in interfacial stereoelectronic effects, confirming the pronounced influence of spatial arrangement on quantum transport (Figure 3b). The study further employed a hovering mode to track random conductance switching trajectories over time in real time, capturing in situ the thermally activated switching kinetics between *cis* and *trans* configurations while observing the regulatory effect of bias electric fields on configurational stability (Figure 3c). This work intuitively demonstrates that SMJs can sensitively resolve variations in electronic state distributions and coupling strengths caused by differences in interfacial spatial orientation, thereby enabling the real-time electrical monitoring of interfacial stereoconformations and their dynamic evolution. Extending this concept to other molecular systems, researchers have further shown that SMJs can capture conductance variations arising from distinct configurational dynamics. For example, a slidable spring-like monomeric molecule was investigated using MCBJ techniques [67]. This molecule simulates intermolecular interactions through intramolecular *π*–*π* stacking, allowing mechanical compression or stretching to modulate conductance over approximately one order of magnitude. The physical origin of this modulation lies in configuration-dependent destructive quantum interference between intramolecular frontier orbitals. In another example, diphenyl disulfide derivatives were manipulated using STM-BJ techniques [68], achieving reversible switching between *π*–*π* interactions and *π*–lone pair interactions by varying the intramolecular dihedral angle. Collectively, these studies demonstrate that SMJs can directly translate subtle molecular configurational changes—such as dihedral angles and stacking distances—into measurable conductance variations.

Building upon these insights into configuration effects, quantifying the steric hindrance of substituents becomes paramount. SMJ conductance measurements have exceptional resolving power for subtle spatial structural variations in anchoring groups, and lay the foundation for the quantitative understanding of interfacial steric effects. The influence of substituent volume in thioether anchoring groups on the transport properties of 4,4′-biphenyl molecular junctions was systematically investigated [69], successfully establishing a quantitative relationship between substituent spatial volume and interfacial electronic coupling. As shown in Figure 3d,e, conductance histograms clearly resolved characteristic conductance peaks corresponding to steric groups ranging from methyl to *tert*-butyl. The conductance of the PP junction for the *tert*-butyl thioether was 1.3 × 10^−4^ *G*_0_, approximately 17 times lower than that of the cyclic locked thioether (2.2 × 10^−3^ *G*_0_). DFT calculations revealed that the Au–S–C–C dihedral angle was constrained to approximately 27° for *tert*-butyl versus 88° for the locked linker, resulting in a tunnel coupling coefficient 4*t*^2^ below 20% of the maximum value. This capability to translate abstract stereochemical effects into quantifiable electrical signatures provides direct experimental evidence for understanding how interfacial steric hindrance precisely modulates electronic coupling strength. By contrast, N-heterocyclic carbene anchoring systems illustrate an alternative scenario regarding steric effects—their substantial spatial volume and rigid molecular structure conversely confer enhanced interfacial stability [71,72], indicating that steric hindrance does not invariably weaken coupling but may instead facilitate the formation of more ordered and stable molecular layers.

Adsorption orientation at interfaces is another critical spatial variable, and SMJs provide a highly sensitive means of detection to track dynamic transitions in such orientations. In one study, the interfacial microenvironment was modulated by varying ionic liquid concentrations [70], employing statistical analysis of single-molecule conductance plateau lengths to directly monitor the continuous evolution of adsorption configurations of 4-(pyridin-4-yl)aniline on gold electrode surfaces (Figure 3f). As the ionic liquid proportion increased, the plateau lengths corresponding to high-conductance states mediated by Au–*π* coupling gradually increased, eventually approaching the characteristic length of upright configurations, whereas plateau lengths for low-conductance states corresponding to Au–*σ* coupling remained stable. This evolutionary map of plateau lengths intuitively reveals a gradual transition in molecule–electrode coupling modes from *π*-dominated tilted adsorption to *σ*-dominated upright adsorption. The nanoscale geometric signatures provided by SMJs serve as sensitive probes for interfacial spatial configuration transitions, enabling real-time observation of how solvent microenvironments drive molecular orientation transitions by modulating electrode electron density, thereby offering intuitive insights into spatial effects at solid–liquid interfaces. Extending this to external field effects, electric-field-induced configurational changes in singly stacked terphenyl molecular junctions were also investigated [73], confirming the fundamental distinction between spatial coupling and chemical bonding. 

### 2.3. Mechanistic Analysis of Interfacial Coupling

The strength and mode of electronic coupling at interfaces directly determine the charge transport efficiency in micro- and nanoscale systems. To gain a deep understanding of these core physical processes, we must systematically resolve the microscopic origins and dynamic evolution of interfacial electronic structures along the dimensions of ideal interface limits, synergistic multiple coupling pathways, and external field modulation.

The interfacial coupling mechanism is fundamentally governed by the relative alignment between molecular HOMO-LUMO levels and the electrode Fermi level, which dictates resonant or off-resonant tunneling transport [74]. Coupling strength is determined by the ratio of the broadening parameter *Γ* to the addition energy *U*_add_: strong coupling (*Γ* ≫ *U*_add_) leads to significant level broadening and partial charge transfer, while weak coupling (*Γ* ≪ *U*_add_) corresponds to incoherent hopping transport. Gate voltage shifts molecular energy levels relative to the Fermi level, enabling HOMO- or LUMO-dominated transport [75]. The image-charge effect pushes both HOMO and LUMO toward the Fermi level, narrowing the energy gap [76]. Electrochemical gating generates a strong electric field via the electric double layer, continuously tuning energy level alignment and inducing redox reactions [77]. Key experimental techniques include inelastic electron tunneling spectroscopy for vibrational fingerprinting [75], mechanically controllable break junctions and scanning tunneling microscope break junctions for statistical conductance measurements [74], and photocurrent and thermopower measurements for determining transport type [78]. Theoretical approaches such as GW-corrected density functional theory and charge-distribution-based corrections accurately describe interfacial polarization effects [76].

**Figure 4:**
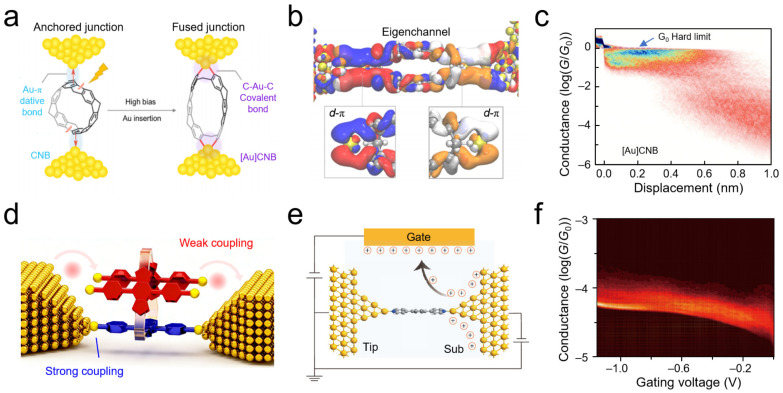
Interfacial coupling regimes and field modulation. (**a**) High-bias C–Au–C bond formation fuses the CNB junction. (**b**) Eigenchannel profile highlights interfacial *d*–*π* hybridization enabling transparent transport. (**c**) Two-dimensional conductance histogram reveals a quantized plateau near *G*_0_, confirming single-channel saturation. Reproduced from Ref. [79]. Copyright 2026 American Chemical Society. (**d**) Schematic illustrating mechanically programmable strong/weak coupling states. Reproduced from Ref. [83]. Copyright 2025 John Wiley and Sons. (**e**) Negative gating induces cation migration, breaking interfacial symmetry. (**f**) Monotonic conductance increase under negative bias confirms chemical potential modulation drives transport in ionic media. Reproduced from Ref. [84]. Copyright 2024 American Chemical Society.

Achieving ideal coupling at metal-molecule interfaces represents a critical topic for exploring the limits of nanoscale electron transport. SMJ techniques provide direct electrical probes for investigating such coupling at the single-bond level. For example, carbon nanobelt molecules form covalent C–Au–C bonds with gold electrodes under high bias, creating atomically fused interfacial structures (Figure 4a). This configuration achieves conductance at the quantum limit *G*_0_ (Figure 4b,c) [79]. At 0.8 V bias, the C–Au–C bonded carbon nanobelt junction exhibited a conductance of *G*_0_ with a clear saturation boundary in the 2D conductance-displacement histogram. Flicker noise analysis also showed that Σ*T*^2^(1–*T*) approached zero near *G*_0_, confirming that the conductance originated from saturation of a single quantum channel with transmission *T* ≈ 1. This work reveals the physical essence of ideal interfacial coupling at the single-chemical-bond level, providing an atomic-scale reference for understanding energy dissipation and transport limits at micro- and nanointerfaces. Furthermore, high conductance approaching the quantum limit was observed through direct Au–π connections between ferrocene molecules and gold electrodes, corroborating the universality of ideal coupling [80]. At 5 K, the most probable conductance of ferrocene during push measurements was 0.017 *G*_0_, and DFT calculations revealed that HOMO-dominated or LUMO-dominated transport depends on the atomic-scale shape of the electrode tip. Similarly, molecular junctions constructed using trimethylstannyl (–SnMe_3_) termini to form Au–C *σ*-bonds also exhibited conductance approaching the quantum limit, with binding energies as high as 3.0 eV, providing another important class of evidence for verifying the universality of transparent interfaces [81,82]. Specifically, the conductance of Au–C bonded alkanes decreased exponentially with a decay constant of 0.97 per methylene, consistent with off-resonant tunneling and a gateway state near the Fermi level. These measurements collectively outline the physical picture of ideal coupling.

By probing multi-channel coupling behaviors, the synergy and competition among different coupling pathways in complex interfaces can be better understood. SMJ techniques enable the in situ resolution of interfacial contact states with different coupling strengths within a single device. Researchers designed molecular junctions with multi-channel parallel configurations, tuning the contact distance between electrodes and anchoring groups through mechanical stretching, thereby achieving continuous modulation of interfacial states from strong to weak coupling on a single platform (Figure 4d) [83]. Three-channel parallel circuits gave conductance states of 10^−3.2^ *G*_0_, 10^−4.1^ *G*_0_ and 10^−4.7^ *G*_0_, with strong coupling channels enhancing conductance 2.5-fold via quantum interference, and weak channels reducing it 13-fold. This strategy effectively eliminates sample-to-sample variations, providing an experimental foundation for establishing quantitative correlations between interfacial coupling strength and charge transport performance. During stretching, the molecular junction sequentially presents fully bound, partially bound, and fully dissociated interfacial contact states, corresponding to three distinct transport modes: high, medium, and low conductance. Electrical characterization confirmed that the high-conductance state originates from quantum coherent transport through three strongly coupled channels, whereas the low-conductance state corresponds to tunneling-dominated mechanisms through three weakly coupled channels, with the intermediate state exhibiting superposition effects of strong and weak coupling channels. Furthermore, utilizing multi-channel coupling effects in π–π stacked dimers, on–off ratios as high as 1,300 were measured, confirming the synergistic regulation by stacking interfacial coupling and intramolecular coupling [15]; molecular junctions based on non-metallic electrodes similarly exhibited multi-channel coupling characteristics in interfacial charge transport [85]. A zinc porphyrin/graphene transistor was used to exploit destructive quantum interference, achieving an ON/OFF ratio >10^4^, subthreshold swing of 14.5 mV/dec at 80 K, and coupling width of *Γ* = 8 meV.

Aside from direct mechanical contact modulation, external fields (such as ionic gating) play equally critical roles in modulating interfacial coupling mechanisms. Ionic liquid gating techniques were employed to observe in situ the reversal of rectification direction in a model system constructed with symmetric electrodes and symmetric molecular backbones [84] (Figure 4e). Studies have revealed that asymmetric ion adsorption at the interface breaking the symmetry of electrode chemical potentials constitutes the primary origin of the rectification effect. The continuous increase in conductance with increasing negative gate voltage directly corroborates this change in interfacial state (Figure 4f). Ionic liquid gating from +1.2 V to −1.2 V fully reversed the rectification direction in symmetric junctions, quantitatively proving that electrode chemical potential modulation dominates level alignment over orbital gating. This SMJ-based measurement reveals the primary mechanisms of interfacial coupling modulation by external fields in ionic liquid environments, providing insights for functional interface design in electrochemical applications. Using three-terminal molecular devices combined with transition voltage spectroscopy (TVS) and inelastic electron tunneling spectroscopy (IETS), the movement of molecular orbitals with gate voltage was observed, demonstrating the evolution of frontier orbitals relative to the electrode Fermi level [86]. Similar systems likewise exhibit modulation of interfacial charge transport by external fields [87]. Furthermore, the modulation effect of bias voltage on van der Waals interfacial coupling was quantitatively characterized by frequency modulation STM break junction (FM-STM-BJ) studies, revealing that increasing bias enhances van der Waals interactions and decreases the conductance attenuation constant, thereby uncovering the microscopic mechanism by which external electric fields influence coupling strength through modification of interfacial electronic structures [59]. Thus, SMJs translate ideal coupling, multi-channel synergy, and field modulation into electrical responses.

The three subsections above have collectively addressed how SMJ measurements reveal the physical nature of interfaces through the perspectives of contact modes, spatial configurations, and electronic coupling mechanisms. These investigations have explored the influence of contact variability, electrode geometry, and electrode material on SMJs. Contact variability manifests as distinct conductance fingerprints that differentiate covalent, coordinate, and van der Waals bonding motifs [55,56]. Electrode geometry, such as single-atom versus multi-atom tip configurations, modulates both the mechanical stability of the junction and the strength of electronic coupling [59,66]. The chemical identity of the electrode/molecule material governs the preferred interfacial bonding type and the resulting charge transport efficiency [69,79,88]. By treating these factors as physically meaningful and quantifiable parameters, the cited works have demonstrated how SMJs leverage these influences to resolve the intrinsic connection between electrode-related characteristics and the junction’s electrical response. The interaction and coupling motifs described above also provide a baseline for understanding how interfaces evolve in the presence of chemical reactions and ion dynamics [89].

## 3. Monitoring of Chemical Interfacial Processes

Section 2 addressed the static physical architecture of interfaces. However, dynamic chemical events that govern real-world functionality, such as bond formation and rupture, catalytic turnover, and ion reorganization usually evolve on microsecond or shorter timescales [36,51], yet ensemble averaging obscures intermediates and rare events during these chemical processes [7,36].

By leveraging high-temporal-resolution conductance–displacement curves, current–time traces, and electrochemical gating techniques [37,38,39,40,41,42,43,90,91], SMJs have enabled significant advances in monitoring covalent bond formation [72,81,82,92,93,94], elucidating catalytic reaction pathways, and probing ion dynamics at solid–liquid interfaces [95,96,97]. Owing to the exceptional sensitivity of SMJs to local electric fields, ionic environments, and mechanical forces at the electrode interface, this platform has emerged as a unique method for investigating chemical reactions within nanoscale confined spaces [91,98,99].

The sections below detail how SMJs capture dynamic chemical events at interfaces. We begin with the in situ monitoring of interfacial covalent bond formation, encompassing the surface reactivity of gold electrodes, radical-mediated coupling, and metal ion-assisted coordination. We then examine the precision with which this approach deciphers reaction mechanisms, specifically by measuring single-bond rupture energies, tracking single-molecule chain assembly, and enabling environment-responsive sensing. Lastly, we address dynamic ionic processes at solid–liquid interfaces, including the identification of specific ion adsorption states, the modulation of local cation distributions within the electrical double layer, and the elucidation of protonation kinetics. Through these time-resolved measurements, SMJs emerge as operando analytical platforms that reveal interfacial chemistry as it happens, with direct implications for catalysis and sensing.

### 3.1. Monitoring of Interfacial Bond Formation

The formation and evolution of interfacial covalent bonds are central to understanding the mechanisms of micro- and nanoscale interfacial chemistry. Serving as the critical bridge connecting molecules to electrodes, interfacial bonding processes directly determine the electronic coupling strength, mechanical stability, and chemical activity of heterogeneous interfaces. In situ characterization of these microscopic bonding events at the nanoscale is essential for elucidating interfacial reaction mechanisms and establishing structure–property relationships at the molecular level.

**Figure 5:**
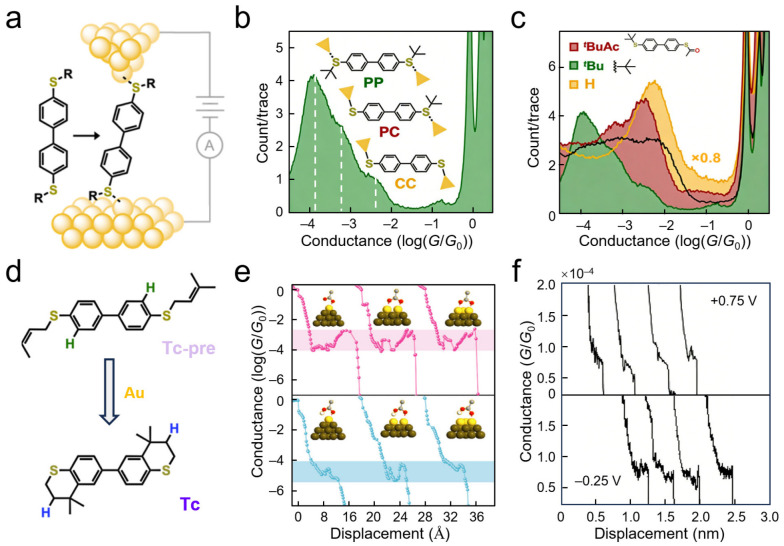
Interfacial chemical processes and bond regulation. (**a**) Schematic of 4,4′-biphenyl-based junctions with thioether-based linkers. (**b**) One-dimensional conductance histogram for the *^t^*Bu molecule. (**c**) Overlaid 1D conductance histograms for *^t^*Bu (green), *^t^*BuAc (red), and H (yellow) references. (**d**) Reaction scheme for the in situ conversion of Tc-pre (light purple) to Tc (dark purple) with proton exchange. Reproduced from Ref. [69] under a CC BY-NC-ND 4.0 license. (**e**) Conductance–stretching distance profiles under varying electrode configurations and protonation states (pink, deprotonated; blue, protonated). Reproduced from Ref. [100]. Copyright 2026 John Wiley and Sons. (**f**) Representative conductance–distance traces at −0.25 V and +0.75 V showing junction formation and breakdown. Reproduced from Ref. [101]. Copyright 2018 American Chemical Society.

Conventional perspectives often regard gold electrodes as relatively inert interfacial materials. However, single-molecule junction experiments have revealed the intrinsic chemical reactivity of gold electrodes during the break junction process. At the single-molecule scale, low-coordination gold surface atoms act as Lewis acid sites, coordinating with sulfur atoms to activate heterolytic S–C bond cleavage and forming Au–S covalent bonds in situ [69] (Figure 5a). Conductance histograms of *tert*-butyl thioether-functionalized molecules exhibit three distinct peaks centered at approximately 10^−5^ *G*_0_, 10^−4^ *G*_0_, and 10^−3^ *G*_0_, corresponding to fully physisorbed, single-sided chemisorbed, and double-sided chemisorbed configurations, respectively (Figure 5b). Notably, the pronounced low-conductance peak arises from substantial steric hindrance imposed by the bulky *tert*-butyl groups, which forces severe deviation of the Au–S bond from the molecular *π*-conjugated backbone, thereby weakening electrode–molecule coupling. Comparative studies of conductance behaviors using alternative anchoring groups, including thioacetyl and cyclic thioether moieties, confirm that the selectivity of this reaction depends on the stability of the leaving group (Figure 5c). Furthermore, experimental observations reveal that gold electrodes can mediate cyclization reactions (Figure 5d), converting dimethyl alkene or tertiary alcohol precursors into cyclic thioether linkages at the interface, with the bonding process tracked in real time through characteristic changes in electrical signals. These findings not only establish gold electrodes as Lewis acid catalysts at the nanoscale but also highlight the capability of SMJs to probe interfacial bond formation with real-time, quantitative precision. In addition to covalent bond formation, gold electrodes also actively participate in reversible coordination switching. For example, amide bond switching within SMJs was utilized to monitor reversible conversions between Au←O and Au←N interfacial configurations in real time, further confirming the dynamic participation of gold electrodes in interfacial chemical evolution [94].

Interfacial chemical processes are influenced by environmental media, particularly proton transfer and water networks. This technique was employed to investigate proton transfer behavior of suberic acid on gold electrode surfaces and its impact on junction conductance [100]. The deprotonation of carboxyl groups proceeds concomitantly with the formation of covalent bonds between oxygen atoms and the gold electrode. Deprotonated molecular junctions exhibit conductance values one order of magnitude higher than their protonated counterparts. Theoretical simulations elucidate conductance plateau characteristics under different electrode configurations (Figure 5e), showing that two-atom electrode configurations significantly reduce the activation barrier for deprotonation. Localized hydronium ion networks in acidic environments accelerate protonation rates, while junction stretching induces reconstruction of the electrode tip from multi-atom to single-atom configurations, driving the interfacial chemical state to switch from deprotonated to protonated. The two-step features observed in conductance trajectories directly reflect dynamic switching of interfacial covalent bond states. This work establishes correlations between solution proton concentration, interfacial proton transfer kinetics, and single-molecule conductance. More broadly, proton-coupled and uncoupled hydrogen-bonding networks were also systematically investigated, revealing the synergistic role of water molecular networks in dynamically modulating interfacial bond states [95]. These studies provide atomic-scale paradigms for understanding the reactivity and dynamic processes of nanoscale metal interfaces in realistic complex environments.

Electrochemical potential is another critical dimension for modulating interfacial bonding stability; it directly determines charge transport efficiency and device lifetime. Break junction techniques were employed to investigate the mechanical stability and electronic coupling of single thiol–gold bonds as a function of electrode potential [101] (Figure 5f). Independent control of tip and substrate potentials enabled the in situ probing of interfacial contact states. Conductance values remained constant within the double-layer charging region, indicating stable electronic coupling strength, whereas mechanical stability exhibits significant potential dependence. Real-time conductance monitoring of SMJs under potential scanning further revealed shortened plateau lengths at both negative and positive potentials, reflecting changes in interfacial binding strength. Positive potentials induce gold oxidation leading to contact rupture, while negative potentials trigger sulfur protonation resulting in weakened bonding. This technique defined the stable potential window for thiol–gold bonds. It has also demonstrated the electrochemical oxidation-mediated conversion of Au←N coordination bonds into highly conductive Au–N covalent bonds, showcasing the application of potential modulation in switching interfacial bonding types [92]. Such investigations reveal the regulatory mechanisms of interfacial chemical reactions on SMJ mechanical properties, providing experimental evidence at the single-bond level for understanding bonding evolution during electrochemical interfacial charge transfer.

To construct specific types of covalent contacts that may go beyond the intrinsic reactivity of gold electrodes, external field-assisted and metal ion-mediated strategies have been introduced into interfacial bonding research, with SMJ platforms functioning as both reactors and detectors.

**Figure 6:**
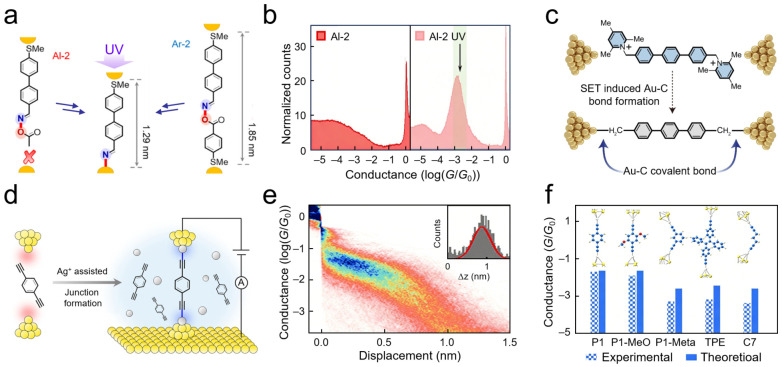
Construction of covalent electrode–molecule contacts. (**a**) Reaction scheme for covalent Au–N bond formation. (**b**) One-dimensional conductance histograms of Al-2 before and after UV irradiation. Reproduced from Ref. [93]. Copyright 2023 American Chemical Society. (**c**) Schematic of electrocatalytic Au–C bond formation in SMJs. Reproduced from Ref. [102] under a CC BY-NC-ND 4.0 license. (**d**) Illustration of the Ag^+^-assisted in situ formation process of Au/Ag–C contacts. (**e**) Two-dimensional conductance–displacement histograms of P1 measured with Ag^+^ additives. (**f**) Experimental versus theoretical Fermi-level conductance values and corresponding optimized junction geometries for alkyne-terminated molecules. Reproduced from Ref. [103]. Copyright 2023 Royal Society of Chemistry.

SMJ techniques can capture highly reactive transient species and resolve interfacial reaction mechanisms in real time, which is particularly suited for radical reactions that are difficult to track using traditional ensemble characterization methods. STM-BJ techniques have been utilized to track the interfacial reaction dynamics of oxime ester derivatives under UV irradiation in real time [93] (Figure 6a). Under dark conditions, oxime ester molecules (Al-2) containing thioether anchoring groups exhibit diffuse conductance signal distributions between gold electrodes, indicating weak electronic coupling between the molecular backbone and electrodes. Following 365 nm UV irradiation, the same molecular system presents a pronounced conductance peak at ~10^−3^ *G*_0_ and corresponding platform extensions in statistical histograms (Figure 6b). This significant change is attributed to specific reactions between iminyl radicals, generated through photolytic homolysis of the oxime ester, and the gold electrode surface, forming covalent Au–N bonded contacts. Electron paramagnetic resonance spectroscopy further validates the generation of iminyl radicals under irradiation, while density functional theory calculations indicate that this bonding process occurs through single-electron bonding between the radical and gold surface atoms, accompanied by the disappearance of radical character. This study demonstrates the capability of SMJs to capture photo-induced interfacial chemical evolution processes, providing direct electrical evidence for resolving interfacial assembly mechanisms in complex environments.

Similarly, electric field-driven electron transfer can serve as an effective means to activate chemical bonds and construct interfacial covalent bonds in situ. Electrocatalytic strategies were employed to achieve single-electron transfer activation of pyridinium scaffolds, generating carbon radicals [102] (Figure 6c). Applied bias drives homolytic C–N bond cleavage, producing highly reactive benzyl radicals that form covalent connections with gold electrode surfaces, thereby achieving in situ construction of sp^3^-type Au–C bonds. Characterizing the conductance confirmed the effective overlap between molecular orbitals and electrodes. This approach reveals microscopic reaction pathways for interfacial bonding, providing clear underlying logic for understanding interfacial chemical processes.

Although gold electrodes can exhibit reactivity under specific conditions, metal ion assistance remains an effective approach for constructing stable covalent metal–carbon interfaces. STM-BJ techniques were employed to observe interfacial bonding processes assisted by silver ions [103] (Figure 6d). In this scheme, silver ions in solution undergo in situ reactions with terminal alkyne molecules, driving the formation of stable Au–Ag–C covalent contacts on gold electrode surfaces. Electrical measurements confirm the construction efficiency of this interface, with two-dimensional conductance histograms in (Figure 6e) displaying clear conductance plateau characteristics, indicating successful fabrication of SMJs. For the alkyne-terminated molecule 1,4-diethynylbenzene, the peak conductance reached approximately 1.0 × 10^−2^ *G*_0_ after Ag^+^ addition, compared to no discernible conductance peak in pure molecule solution, demonstrating that the Ag^+^-assisted method enables the formation of well-defined single-molecule junctions. The introduction of silver ions significantly enhances junction formation probability and is applicable to various conjugated and saturated molecular scaffolds (Figure 6f). This study directly captures the bonding process and critical information regarding the resulting interfacial electronic coupling strength, further demonstrating the unique advantages of SMJs as nanoprobes for resolving interfacial chemical kinetics.

### 3.2. Precise Analysis of Chemical Reactions

The dynamic evolution of interfacial chemical reactions constitutes the central element governing the functionality of micro- and nano-scale interfaces. Elementary reaction steps at interfaces involve the generation of transient intermediates, bifurcation of reaction pathways, and microscopic mechanisms of energy dissipation. Precise resolution of the real-time kinetics and microscopic mechanisms underlying these interfacial reactions provides a crucial foundation for establishing theoretical frameworks of interfacial reactivity and enabling rational control over interfacial functions.

**Figure 7:**
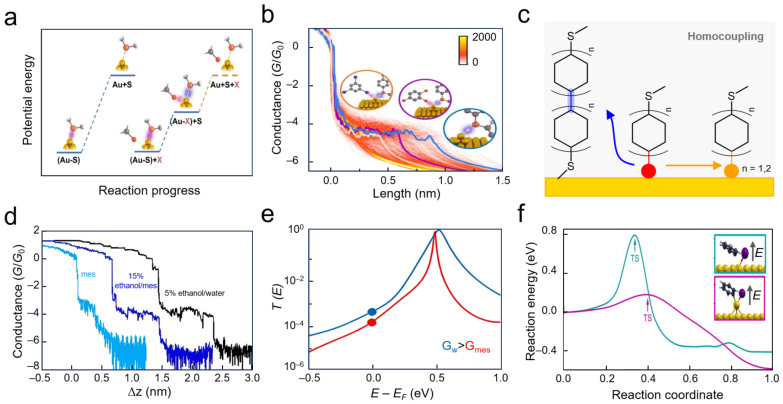
Interfacial bond energetics, reaction pathways, and microenvironmental effects. (**a**) Schematic illustration of the reduced energy barrier for Au–S bond cleavage under halogen catalysis. (**b**) Statistical distributions of plateau length and conductance under varying catalytic conditions. Reproduced from Ref. [104] under a CC BY-NC-ND 4.0 license. (**c**) Reaction pathways for interfacial Au–C bond formation (orange) and C–C coupling (blue) of tributyltin-terminated molecules. Reproduced from Ref. [105]. Copyright 2024 American Chemical Society. (**d**) Logarithmic conductance–distance retraction curves of the devices in different environments. (**e**) Calculated transmission functions of the device in different environments (blue, water; red, mesitylene). Reproduced from Ref. [106]. Copyright 2021 John Wiley and Sons. (**f**) Energy profile of iodobenzene dehalogenation on Au(111) with/without a Au adatom (green, without; pink, with), showing electric-field effects. Reproduced from Ref. [107] under a CC BY-NC 3.0 license.

Interfacial bonding strength can be quantitatively analyzed by correlating electrical signals with chemical bond energies during junction stretching. For example, a high-resolution STM-BJ method was used to establish a quantitative link between conductance plateau duration and bond rupture energy barriers [104]. Specifically, halogen atoms acting as catalysts lower the Au–S bond rupture energy, accelerating bond cleavage and shortening plateau lengths. Compared to catalyst-free conditions, chlorinated solvents produced shorter plateaus, and iodobenzene even shorter ones, directly distinguishing chlorine and iodine catalytic activity at the single-bond level (Figure 7a). For these junctions, the most probable plateau length was 0.97 nm in dodecane, 0.59 nm in trichlorobenzene, and 0.46 nm after adding triiodobenzene, corresponding to Au–S bond cleavage energies of approximately 1.5 eV, 1.0 eV, and 0.5 eV, respectively. AFM force spectroscopy confirmed that the rupture force decreased from about 0.8 nN in dodecane to about 0.6 nN in trichlorobenzene. Further theoretical calculations confirm that halogens stabilize reaction intermediates, reducing the activation energy for bond rupture (Figure 7b). This work reveals catalytic reactions from a single-bond energy perspective, offering a bottom-up view of macroscopic interfacial catalysis. 

Such detection capabilities extend beyond bond rupture to encompass real-time monitoring of complex synthetic processes such as molecular coupling. Taking tributyltin-terminated aromatic molecules as an example, the STM-BJ technique can capture stepwise chemical reaction pathways on gold electrode surfaces under weak electric field conditions [105] (Figure 7c). When molecules bridge between gold electrodes, cleavage of the terminal tin–carbon bond forms a high-conductance Au–C covalent contact, while carbon–carbon homocoupling occurs simultaneously at adjacent active sites to generate biphenyl dimers characterized by significantly lower conductance values compared to monomers, accompanied by noticeably increased junction elongations. This system achieves efficient C–C coupling at ultra-low bias voltages of approximately 5 mV, with reaction efficiency changing markedly as solvent dielectric constant varies; high-dielectric environments promote coupling product formation, a phenomenon subsequently confirmed through off-line high-performance liquid chromatography validation. 

When the molecular scaffold extends to biphenyl systems, coupling reactions can generate quarterphenyl; however, introducing sterically bulky substituents or additional auriphilic groups into the molecular backbone effectively suppresses bimolecular coupling pathways through steric hindrance effects, preserving only the single-molecule Au–C bonding channel. Complex chemical reaction pathways and kinetics were tracked in real time using electrical signal analysis, providing direct evidence for microscopic mechanisms and regulatory factors governing solid–liquid interfacial chemical reactions. 

The occurrence and evolution of interfacial chemical reactions depend profoundly on the local microenvironment. The sensitive dependence of electron transport on external chemical environments provides the physical basis for investigating specific reactions. For example, medium variations can modulate molecular orbital alignment relative to electrode Fermi levels, shifting single-molecule conductance (Figure 7d) and transmission resonances (Figure 7e) [106]. Beyond such physical modulation, in aqueous or protic solvents, water networks and protons/metal ions synergistically induce pH- and ion-concentration-dependent conductance responses. The stable-state transitions observed reflect the microscopic kinetics of protonation–deprotonation equilibria and ion coordination–dissociation processes. This real-time tracking capability enables identification of transient intermediates and investigation of reaction mechanisms in complex environments [99].

Leveraging the unique tunneling-sensitive mechanism, single-molecule techniques can further enable the real-time identification of reactive intermediates and products, validating the effectiveness of molecular junction devices in resolving the chemical composition and dynamic evolution of solid–liquid interfaces during reactions [108]. Single-molecule junction platforms based on metal electrode tunneling effects have thus been extended to the in situ detection of chemical species generated at electrode surfaces [109]. Stone and colleagues employed STM-BJ to identify product formation in aryl iodide Ullmann coupling on rough gold surfaces in real time. Low-coordination Au sites on rough gold surfaces significantly reduce the activation energy for aryl iodide dehalogenation/oxidative addition, while local interfacial electric fields further modulate this energy barrier, theoretically revealing the synergistic catalytic effect of gold/liquid interfaces on Ullmann coupling reactions (Figure 7f) [107]. Additionally, SMJs have been employed to investigate how external electric fields modulate chemical reaction pathways near interfaces [91,98].

### 3.3. Monitoring of Interfacial Ion Dynamics

The distribution and dynamic transport of ions at solid–liquid interfaces constitute crucial elements in regulating interfacial functional properties. Specific adsorption of ions on electrode surfaces, spatial arrangement within the electric double layer, and interactions with interfacial functional groups profoundly modulate local electric field distributions, molecular energy level alignments, and activation barriers for interfacial reactions. These microscopic dynamic processes involve not only transitions in physical adsorption configurations but also direct participation in complex elementary interfacial reactions such as proton-coupled electron transfer (PCET). Precisely resolving the distribution patterns and dynamic evolution pathways of ions at interfaces, and quantitatively revealing the microscopic kinetic mechanisms underlying their participation in interfacial reactions, are essential foundations for understanding the nature of solid–liquid interfaces and establishing theories of interfacial ion dynamics.

**Figure 8:**
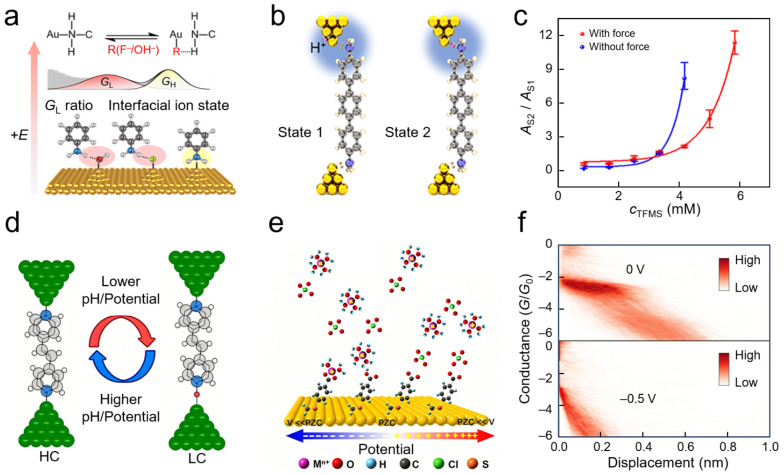
Electrical probing of interfacial ionic states and double-layer dynamics. (**a**) Schematic of the anion-adsorbed interfacial state based on an amine-anchored probe. Reproduced from Ref. [110]. Copyright 2024 American Chemical Society. (**b**) Protonated and deprotonated interfacial states alongside their corresponding single-molecule configurations. (**c**) Influence of proton concentration and external mechanical force variations on interfacial proton transfer. Reproduced from Ref. [111] under a CC BY-NC-ND 4.0 license. (**d**) Illustration for real-time monitoring of single-molecule protonation at the molecule–metal interface. Reproduced from Ref. [112]. Copyright 2018 American Chemical Society (**e**) Model of interfacial coordination/contact states mediated by cations. (**f**) Two-dimensional conductance–displacement statistical histogram demonstrating the cation-induced reversible modulation of interfacial switching at various applied potentials. Reproduced from Ref. [114] under a CC BY-NC-ND 4.0 license.

Understanding solid–liquid interfacial structures begins with clarifying ion adsorption configurations on electrode surfaces and their dynamic responses to external stimuli. Wei and co-workers constructed a single-molecule probe system based on amino anchoring groups, utilizing interactions between specific anions and active hydrogen atoms of amino groups to detect interfacial ionic states [110]. Theoretical calculations indicated that the binding energy of anions on gold electrode surfaces is significantly higher than that of gold–nitrogen coordination bonds, driving the formation of interfacial adsorption states (Figure 8a). Experimentally, two conductance states—high and low—were observed, corresponding, respectively, to gold–nitrogen coordination-dominated and ionic hydrogen bond-involved interfacial coupling modes. The authors subsequently employed noise power spectral density analysis to confirm that the low-conductance state originates from a synergistic transport mechanism involving both through-space and through-bond pathways. Applied electric fields can alter surface charge densities on electrodes, thereby modulating local anion concentrations and the probability of ionic hydrogen bond formation. This real-time monitoring at the single-bond level reveals the dynamic response patterns of ion distributions at solid–liquid interfaces under external fields. 

Along with anion adsorption, interfacial proton behavior represents another critical component of ionic processes at solid–liquid interfaces. STM-BJ technology was employed to achieve real-time electrical monitoring of single-event protonation processes at metal–liquid interfaces, directly capturing reversible switching between unprotonated and protonated states of amino terminal groups and quantitatively revealing the regulatory effects of interfacial proton concentration on protonation/deprotonation kinetics (Figure 8b). Simultaneously, applied mechanical force could further influence proton insertion processes between molecular anchoring groups and metal electrodes, indicating that interfacial proton transfer exhibits not only significant microenvironment dependence but also active regulation by external fields (Figure 8c). This work provides direct evidence for understanding proton behavior and its dynamic regulation mechanisms at solid–liquid interfaces from a single-molecule perspective [111].

Additionally, interfacial proton behavior in Ni/4,4′-vinylene dipyridine/Ni SMJs was investigated using electrochemically controlled STM-BJ techniques. It was discovered that molecular conductance could be switched synergistically by solution pH and applied potential. High and low conductance states were attributed to unprotonated and protonated pyridine anchoring states, respectively (Figure 8d), while real-time electrical capture of protonation/deprotonation processes at electrode–molecule interfaces was achieved [112]. This work demonstrates that proton behavior at solid–liquid interfaces not only reshapes metal–molecule contact modes but also manifests as directly readable dynamic proton transfer processes at the single-molecule scale. Similarly, a positively charged electrostatic anchoring strategy was utilized to achieve sensitive detection of the interfacial ionic environment through non-covalent interactions between pyridinium groups and gold electrodes, discovering that different counter-anions could induce significant conductance differences [113]. Furthermore, guest molecules with different charge states (negative, positive, neutral) were introduced into permethylated *α*-cyclodextrin cavities, revealing how ionic charge in the local microenvironment modulates interfacial properties via host–guest interactions [97].

While identification of anion adsorption states provides a foundation for understanding interfacial behavior, the distribution of cations within the electric double layer further determines the coupling properties between molecules and electrodes in local microenvironments. SMJs were employed to probe the chemical regulation of interfacial contacts by localized cations within the outer Helmholtz plane [114]. Under negative potential, hydrated metal cations coordinate with carboxylate groups, suppressing direct gold–carboxylate contact (Figure 8e). Conductance measurements showed switching behavior: at zero potential, prominent conductance peaks and stretching plateaus indicated the ON-state (Figure 8f); at −0.5 V, signals dropped to background, corresponding to the OFF-state. The ON-state conductance was approximately 1.0 × 10^−3.0^ *G*_0_, while the OFF-state conductance fell below the detection limit of 10^−7.0^ *G*_0_, yielding an ON/OFF ratio exceeding 10^4^. These variations arise from cation dynamics and coordination chemistry [115]. SMJs thus reveal ion–functional group interactions at interfaces. In another study, the authors designed a pyridinium salt guest molecule containing an azobenzene group that forms a host–guest complex with permethylated α-cyclodextrin [116]. Under alternating visible and ultraviolet irradiation, they achieved reversible switching between bimodal and trimodal conductance states, where photo-induced charge distribution changes directly influenced the interfacial ionic atmosphere.

Ion distribution and coordination at interfaces not only affect physical coupling but also directly participate in complex chemical reaction processes. SMJ techniques can resolve complex reaction kinetics involving ion transfer at solid–liquid interfaces. In situ monitoring of reaction processes in a benzothiadiazole molecular catalytic hydrogen evolution system was achieved using an electrochemical mechanically controllable break junction (EC-MCBJ) platform [117]. This work demonstrates the unique advantages of SMJs in gaining a deep understanding of microscopic mechanisms of solid–liquid interfacial reactions. Such studies indicate that SMJs can probe deep into the microscopic mechanisms of interfacial reactions, providing quantitative experimental evidence for understanding quantum effects in ion dynamic processes. Furthermore, utilizing SMJ platforms, researchers can in situ resolve proton-coupled electron transfer behaviors in hydrogen-bonded interfaces and the influence of charge-assisted hydrogen bonding on electron transport, thereby deepening our understanding of noncovalent interfacial microscopic mechanisms [37,95].

**Figure 9:**
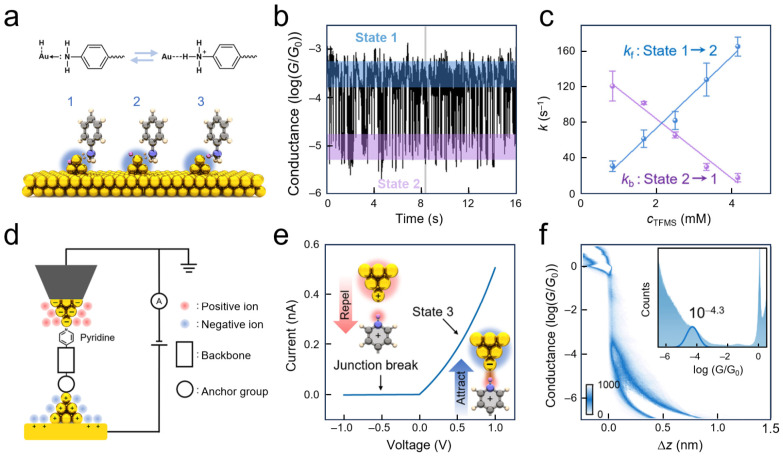
Electrical tracking of single proton reaction dynamics at the interface. (**a**) Schematic illustration of single-event protonation/deprotonation processes. (**b**) Real-time conductance–time traces of stochastic two-state transitions between protonated and deprotonated states. (**c**) Dependence of forward and reverse rate constants on the proton concentration within the interfacial microenvironment. Reproduced from Ref. [111] under a CC BY-NC-ND 4.0 license. (**d**) Model of interfacial states at the pyridine molecule/ion–gold electrode interface. (**e**) Stabilization and rupture behaviors of molecular junctions driven by interfacial electrostatic interactions. (**f**) Transformation characteristics of interfacial states associated with proton insertion. Reproduced from Ref. [118] under a CC BY-NC 4.0 license.

Macroscopic reaction rates originate from the kinetic behavior of microscopic particles. Building upon the static identification of interfacial ion adsorption states, SMJs also demonstrate potential for resolving microscopic particle dynamics. Utilizing the hover mode of STM-BJ techniques, amino-terminated molecules fixed between gold electrodes can serve as probes for interfacial protonation reactions, capturing reversible switching of single-event protonation and deprotonation through real-time conductance–time trajectories [111] (Figure 9a). Bimodal conductance signals randomly switch on the millisecond timescale (Figure 9b), and after idealization using hidden Markov models, residence time distributions for protonated and neutral states can be extracted. This system provides deep insights into how interfacial microenvironments regulate reaction rates. As shown in Figure 9c, although increased proton concentration enhances interfacial proton density to promote protonation, it simultaneously inhibits deprotonation due to solvation effects arising from local charge enrichment. This results in a positive linear correlation between the forward reaction rate constant and proton concentration, but a negative linear correlation for the reverse reaction rate constant. Such time-resolved electrical measurements not only enable the monitoring of interfacial proton transfer kinetics at the single-event level, but also provide direct evidence linking macroscopic electrochemical phenomena with microscopic molecular mechanisms. In broader kinetic studies, novel single-molecule devices were utilized to monitor the formation and dissociation kinetics of host–guest complexes in real time [43], extracting binding and dissociation rate constants and their temperature dependencies from current–time trajectories, demonstrating the versatility of SMJs in measuring interfacial reaction kinetics.

To further elucidate microscopic mechanisms of interfacial protonation reactions with single-bond precision, later research focused on identifying reaction intermediates and tracking their transformation pathways, particularly under external force fields. STM-BJ techniques were utilized to construct a solid–liquid interface model system between pyridinium ions and gold electrodes, achieving real-time monitoring of proton-involved interfacial reaction processes at the single-molecule level [118]. As shown in Figure 9d, by regulating the solution–electrode interface electrostatic potential, the system forms an ion/metal interfacial state comprising terminal group–hydrogen–gold. The existence of this unique interfacial state provides a structural foundation for studying reversible protonation reactions. Electrical characterization revealed that under positive bias, gold electrode tips accumulate negative charges, generating electrostatic attraction with protonated pyridinium ions to maintain junction stability; whereas under negative bias, the electrode surface carries positive charges, producing electrostatic repulsion with the positively charged ions that leads to junction rupture and current disappearance (Figure 9e).

Furthermore, by stretching molecular junctions at different rates, conductance plateau lengths and lifetimes were quantitatively compared before and after protonation. The results indicated that proton insertion is limited by proton diffusion, whereas rupture of the charged interfacial state depends primarily on electrode distance (Figure 9f). This research reveals kinetic details of proton insertion under external force modulation and provides single-bond evidence for ion transport and reaction mechanisms at electrochemical interfaces. Through such detection strategies, interfacial reactions in macroscopic electrocatalytic and energy storage systems can be resolved at the molecular level.

Additionally, the frequency-modulated STM-BJ techniques used in the aforementioned work to quantitatively characterize bias modulation effects on van der Waals interfacial coupling [59] can also be used to quantify dynamic processes in ionic atmospheres at interfaces. Polarity-reversible molecular rectifiers were built using charge-transfer complexes [119], revealing ion-gating-induced switching of transport mechanisms from HOMO-dominated to LUMO-dominated, providing experimental evidence for understanding how electric field-induced interfacial ion redistribution affects electron transport.

## 4. Conclusions and Perspectives

The macroscopic physicochemical properties of material interfaces are rooted in their microscopic structures and dynamic behaviors at micro/nanoscales. By introducing recent advances in resolving physicochemical processes at micro/nano interfaces, this review systematically elaborates the unique value of SMJs as a detection platform for interfacial processes.

Regarding the analysis of interfacial physical properties, SMJs enable precise discrimination of interfacial contact modes. Through electrical measurements including conductance fingerprints and noise spectroscopy, different types of interfacial interaction—such as covalent bonds, coordination bonds, and van der Waals forces—can be clearly distinguished, and their dynamic evolution processes captured. Furthermore, single-molecule techniques have atomic-scale spatial resolution capabilities, allowing the probing of subtle steric effects—including substituent volume, molecular configuration, and adsorption orientation—on the modulation of electronic coupling. At the level of interfacial coupling mechanisms, single-molecule platforms provide pristine research models ranging from ideal quantum-limit contacts and multi-channel cooperative transport to dynamic regulation of energy levels and coupling strength by external fields (electric fields, ionic gating), thereby deepening the understanding of the essential nature of charge transport across interfaces.

In terms of monitoring interfacial chemical processes, SMJs have propelled interfacial chemistry research from static structural description to real-time reaction tracking. They enable in situ monitoring of the formation kinetics of covalent bonds such as Au–C and Au–S at electrode interfaces, capturing transient reaction intermediates including photoinduced radicals and electrochemically activated species. More importantly, these techniques can resolve microscopic reaction pathways and single-bond energetics, such as distinguishing catalytic activity differences among halogen atoms and tracking stepwise processes of C–C coupling. For the complex environment of solid–liquid interfaces, SMJ probes can identify adsorption states of specific anions, detect local distributions of cations within the electric double layer and their modulation of molecular coupling, and delve into elementary kinetics and quantum tunneling mechanisms involving ionic processes such as proton transfer and hydrogen evolution reactions.

SMJ techniques have transcended their initial focus on molecular electronics to emerge as a pivotal methodology that bridges macroscopic interfacial performance with microscopic physicochemical mechanisms. This evolution has yielded profound insights into nanoscale interfacial science and is now steering the field toward more dynamic and application-oriented paradigms. Looking ahead, realizing this potential will require the synergistic integration of multidimensional characterization and intelligent data analytics. Future SMJ platforms are expected to converge electrical, optical, mechanical, and thermal probes into unified setups, enabling the simultaneous and correlated monitoring of multiple physicochemical parameters at the single-molecule level. Such multimodal architectures are essential for deciphering previously inaccessible coupling pathways and energy dissipation mechanisms that govern interfacial behavior.

Concurrently, the increasing complexity and volume of experimental data will necessitate the adoption of machine learning and artificial intelligence for high-throughput analysis [120]. These tools are highly effective for managing break-junction datasets: supervised CNNs classify conductance traces to discriminate interfacial contact configurations [121,122], whereas unsupervised clustering reveals hidden subpopulations and rare quantum-transport behaviors [123,124,125]. Coupled with denoising techniques that recover transient interfacial states [126] and hybrid strategies overcoming the limitation of sparse data labeling [127], these approaches are steering SMJ investigations toward automated, real-time analysis of interfacial physicochemistry. By coupling multimodal experimentation with AI-driven analytics, SMJ research will progressively shift from static structural characterization toward real-time dynamic regulation. This methodological maturation will further propel investigations from idealized model systems into complex, practical environments spanning catalysis, energy conversion, and molecular sensing.

Ultimately, the convergence of these technical and analytical advances will cement SMJ techniques as an indispensable bridge connecting the macroscopic functionality of nanomaterials with their fundamental interfacial nature. This integrated approach not only promises to unravel the underlying physicochemical principles of nanoscale systems but will also establish a robust scientific foundation for the rational design, accurate function prediction, and scalable application of next-generation functional interfacial materials.

## Data Availability

No new data were created or analyzed in this paper.

## Abbreviations

The following abbreviations are used in this manuscript:

SMJ(s)	Single-molecule junction(s)
MCBJ	Mechanically controllable break junction
STM-BJ	Scanning tunneling microscope break junction
CAFM-BJ	Conductive atomic force microscope break junction
*G*_0_	Quantum conductance unit
FM-STM-BJ	Frequency-modulated scanning tunneling microscope break junction
TVS	Transition voltage spectroscopy
IETS	Inelastic electron tunneling spectroscopy
PCET	Proton-coupled electron transfer
HOMO/LUMO	Highest occupied/lowest unoccupied molecular orbital
EC-MCBJ	Electrochemical mechanically controllable break junction
